# Thermal Shock-Activated Spontaneous Growing of Nanosheets for Overall Water Splitting

**DOI:** 10.1007/s40820-020-00505-2

**Published:** 2020-08-12

**Authors:** Han Wu, Qi Lu, Jinfeng Zhang, Jiajun Wang, Xiaopeng Han, Naiqin Zhao, Wenbin Hu, Jiajun Li, Yanan Chen, Yida Deng

**Affiliations:** 1 School of Materials Science and Engineering, Tianjin Key Laboratory of Composite and Functional Materials, Key Laboratory of Advanced Ceramics and Machining Technology, (Ministry of Education), Tianjin University, Tianjin, 300350 People’s Republic of China; 2 Joint School of National University of Singapore and Tianjin University International Campus of Tianjin University, Binhai New City, Fuzhou, 350207 People’s Republic of China

**Keywords:** Ultrafast synthesis, Spontaneous growing, Thermal shock, Seed inducing, Water splitting

## Abstract

**Highlights:**

- Nanomaterials-based nickel foam (NF-C/CoS/NiOOH) with nanosheets structure and core–shell heterostructure was prepared for the first time by a facile and fast synthesis strategy of Joule-heating and water soaking treatment.
- The formation mechanism of nanosheets structure was proposed that the driving force of nanosheets structure generation was the metastable nickel activated by thermal shock, and the CoS could induce the NiOOH nanosheets growing continually.
- NF-C/CoS/NiOOH exhibited good oxygen evolution, hydrogen evolution, and overall water splitting performance.

**Abstract:**

Nanomaterials based on nickel foam (NF) have been widely applied in energy conversion and storage field. Traditional synthesis methods such as hydrothermal method which is dangerous and high cost limited the scalable developments. Herein, we report a fast, simple, and low-cost synthesis method of nanomaterials based on NF by Joule-heating and water soaking treatment. Thin carbon-coated CoS on NF (NF-C/CoS) was synthesized by Joule-heating for a few seconds with rapid cooling. And then, NF-C/CoS/NiOOH with core–shell heterostructure was fabricated by soaking treatment of NF-C/CoS in water on which NiOOH nanosheets grew spontaneously. The formation mechanism is proposed that the coordination complex precursor converts into C/CoS on NF driven by Joule-heating, and the nickel on the surface of NF is activated to form metastable nickel simultaneously. The metastable nickel reacting with water leads to the formation of NiOOH, and the induction of CoS makes NiOOH grow continuously. This synthesis technology provides a new route to manufacture NF-based nanostructures, and the as-fabricated NF-C/CoS/NiOOH exhibits great potential as electrocatalyst for oxygen evolution reaction and hydrogen evolution reaction.
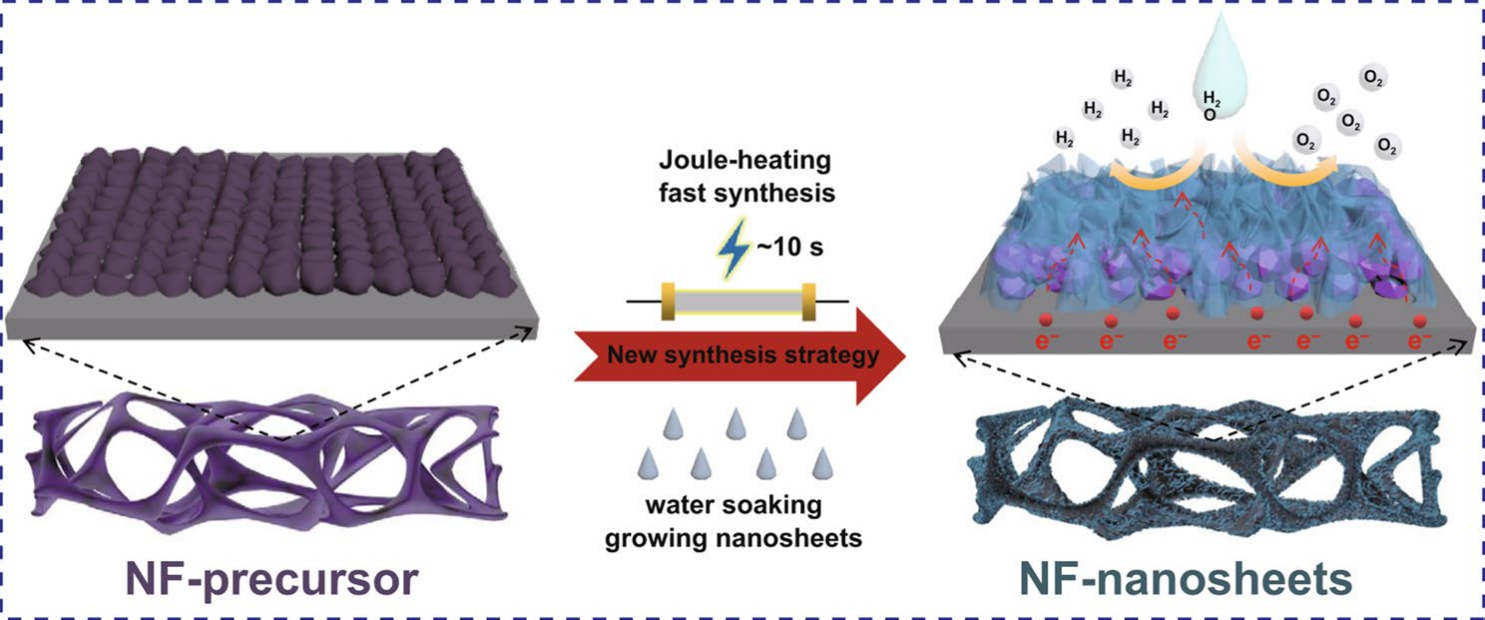

**Electronic supplementary material:**

The online version of this article (10.1007/s40820-020-00505-2) contains supplementary material, which is available to authorized users.

## Introduction

Sustainable and green energy resources are becoming extremely important to meet the growing demand for energy developments due to the ever-rising environmental pollution and the increasing consumption of fossil fuels [1]. Recent studies on developing sustainable energy strategies to address the current environmental and energy challenges have attracted significant interests [2]. Electrocatalytic water splitting including hydrogen and oxygen producing is widely regarded as the most economical and effective way to produce clean and sustainable energy [3]. The overpotential corresponding to thermodynamics and kinetics of oxygen evolution reaction (OER) and hydrogen evolution reaction (HER) which are the half-reaction of water splitting depends on electrocatalytic materials [4].

Noble metal electrocatalysts such as Pt, Ru, and Ir have superior OER and HER electrocatalytic activities, but the development of noble metal electrocatalysts is restricted by the high-cost and conventional coating method in which the binder increases the resistance, buries the active sites, inhibits mass/electrons transports and the loading mass of the electrocatalysts is usually less than 1 mg cm^−2^, providing limited catalytically active sites [5, 6]. Enormous efforts have been dedicated to the development of effective non-noble electrocatalysts and self-supporting electrode [7]. Nanomaterials based on nickel foam (NF) as self-supporting electrocatalysts have been extensively studied because NF as a high conductive substrate has the porous structure to provide higher active area, and nickel has good electrocatalytic activity. The NF which is widely used in practical applications such as NiMH battery [8], fuel cells [9], and electrocatalysts [10, 11] is low-cost and mature in industrial production. Studies in recent years have shown that NF-based nanoarchitectures as electrocatalysts are mainly prepared by hydrothermal or solvothermal methods because nickel has high reactivity with other additives to form kinds of nanostructures such as nanosheets and nanorods in water or other organic solvents under high temperature and pressure [12, 13]. However, hydrothermal and solvothermal processing requires high temperature and high pressure lasting a long time, which are harsh and dangerous conditions during industrial manufacturing. The use of additives and organic solvents increases the cost and may be harmful to the environment. These are not conducive to the industrial production of electrocatalysts based on NF. Therefore, it is necessary to find a fast and low-cost method to manufacture NF-based electrocatalysts. In 2016, Chen et al. for the first time reported nanoparticles synthesized by simple Joule-heating and rapid quenching, which provide a new method of ultrafast nanomanufacturing [14]. In the next few years, Yao et al. prepared high-entropy-alloy nanoparticles and single atoms loaded carbon nanofibers for small molecule electrocatalysis in the same method [15, 16]. Chen et al. prepared FeS_2_ nanoparticles on reduced graphene oxides from FeS_2_ microparticles in the same method [17]. This method which is essentially a thermal synthesis processing has superfast synthesis speed, but it still has limitations such as too high synthesis temperature and over-reliance on carbon supports.

**Fig. 1:**
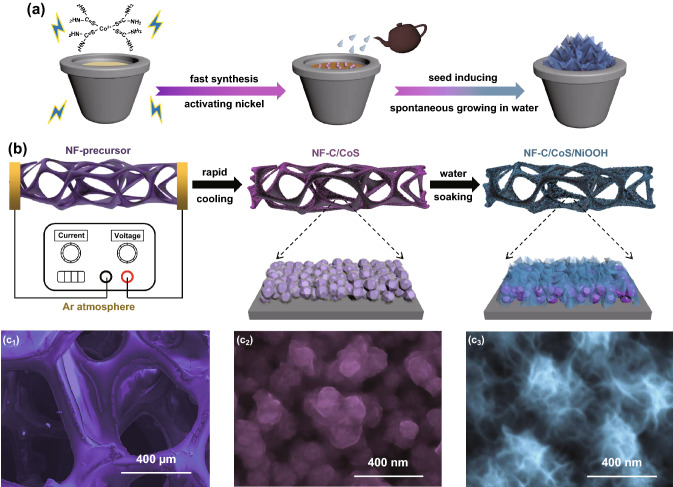
**a** Synthesis strategy of NF-C/CoS/NiOOH. **b** Schematic illustration for preparation of NF-C/CoS/NiOOH. SEM of **c**_**1**_ NF-precursor, **c**_**2**_ NF-C/CoS and **c**_**3**_ NF–NF-C/CoS/NiOOH

Here, we designed and fabricated an integrated electrocatalyst (NF-C/CoS/NiOOH) by using NF as Joule-heating substrate and water soaking treatment innovatively. The cobalt–thiourea coordination complex as precursor loading on NF in situ transforms into doped carbon-coated CoS (NF-C/CoS) by Joule-heating with raped cooling which leads to nickel activated to be metastable that is like the “seeds” forming magically and the “soil” becoming fertile through lightning (Fig. 1a, b). It is remarkable that NF-C/CoS/NiOOH with nanosheet structure was fabricated by simple soaking treatment of NF-C/CoS in water on which a nanostructure formation mechanism was proposed for the first time (Fig. 1b). The metastable nickel reacting with water led to the formation of NiOOH nanosheets and the C/CoS like “seeds” in the “flowerpot” induced flowerlike NiOOH nanosheets growing spontaneously and continuously (Fig. 1a). NF-C/CoS/NiOOH exhibited good OER, HER, and overall water splitting electrocatalytic performance.

## Experimental Section

### Materials Preparation

#### Chemicals

Cobalt (II) acetate tetrahydrate, thiourea, Pt/C (wt % = 20%), and IrO_2_ were purchased from Aladdin (Shanghai, China). Nickel foam (NF) without any treatment was cut to 3.5 cm × 0.5 cm × 0.1 cm.

#### Preparation of NF-C/CoS/NiOOH

Cobalt (II) acetate tetrahydrate and thiourea were dissolved into ethanol to prepare 1 M cobalt–thiourea coordination complex solution. The solution was dropped into the NF with 100 μL cm^−2^ and then the NF dried at 60 °C for 30 min. The direct current supply was adopted for Joule-heating (MAISHENG-MP1203D, 0–50 A, 0–50 V). The as-prepared cobalt–thiourea coordination complex solution was dropped into the nickel foam as shown in Fig. R3. The distance between powered clamps was 2.5 cm. The cobalt–thiourea coordination complex loading nickel foam clamped on the power clamps was transferred into the argon glove box. The clamp was passed to a current of 5A for 10–15 s, when the nickel foam changes from purple to black (Fig. S1b, c). Finally, the NF-C/CoS/NiOOH was prepared by soaking NF-C/CoS in water overnight.

#### Preparation of NF-C/Ni(OH)S

Thiourea was dissolved into ethanol to prepare 1 M solution. The solution was dropped into the NF with 100 μL cm^−2^ and then the NF dried at 60 °C for 30 min. The NF clamped on the power clamps was passed a current of 5 A in the argon glove box for about 10 s and then soaking in water overnight.

#### Preparation of Comparative Samples

In order to prove the formation processing of NF-C/CoS/NiOOH, a series of comparative samples were prepared. The sample NF-5A was prepared by same as NF-C/CoS/NiOOH without the dropping of the cobalt–thiourea coordination complex solution. The sample NF-C/CoS/NiOOH-5 min was prepared by only changing the soaking time to 5 min. The sample NF-cobalt oxide was prepared by same as NF-C/CoS/NiOOH with only cobalt–ethanol solution. The samples named NF-C/CoS/NiOOH-3A, NF-C/CoS/NiOOH-7A, NF-C/CoS/NiOOH-10A were prepared by only changing the current from 5 to 3, 7, and 10 A without other changes in the synthesis of NF-C/CoS/NiOOH.

### Materials Characterization

The microstructure, energy-dispersive X-ray spectroscopy (EDS), and element distribution mapping were characterized by scanning electron microscopy (SEM, JSM-7800F), transmission electron microscopy (TEM, JEOL-2100F). Raman spectra were measured using Edinburgh RM5 with the excitation laser line at 532 nm. Light microscopy images were characterized by using SOPTOP-BH200m. X-ray photoelectron spectra test was performed on a scientific ESCALAB 250 instrument. The temperature evolution during the synthesis of NF-C/CoS was performed using an ImageIR8355BB high-speed thermal imaging camera.

### Electrochemical Measurement

Electrochemical measurements were taken in a three-electrode system on an electrochemical workstation (CH Instruments 660E) at room temperature in 1 M KOH solution. NF-C/CoS/NiOOH, NF-C/CoS and NF (0.5 × 2 cm^2^) were used directly as the working electrode. A graphite rod was used as the counter electrode and saturated calomel electrode (SCE) was used as the reference electrode. 10 mg IrO_2_ and 10 mg Pt/C were dispersed, respectively, into 965 μL isopropyl alcohol and 35 μL Nafion solution (5%) with 30 min sonication. NF-Pt/C and NF-IrO_2_ were prepared by loading powder ink (200 μL) onto NF. Alkaline overall water splitting measurement was taken in a two-electrode system by using bifunctional NF-C/CoS/NiOOH as both anode and cathode in 1 M KOH. The measured potentials were normalized to reversible hydrogen electrode (RHE) based on the Nernst equation:

1 $$E\left( {\text{RHE}} \right) = E\left( {\text{SCE}} \right) + 0.059 \times {\text{pH}} + 0.24.$$

All the polarization curves were tested at a scan rate of 5 mV s^−1^. All the polarization curves and chronopotentiometry curves were corrected for iR losses. Electrochemical impedance spectroscopy (EIS) were measured in a frequency from 10^5^ to 0.01 Hz at 1.526 V (OER) vs RHE, − 0.173 V (HER) vs RHE and 1.72 V (overall water splitting). The Faraday efficiency of OER and HER on NF-C/CoS/NiOOH was tested and analyzed by using gas chromatograph (GC) with gas-tight H-cell. Employ a GC, equipped with a combination of molecular sieve 5 A (2 m × 4 mm), Porapak-N (2 m × 4 mm), and Porapak-N (3 m × 4 mm), for O_2_ and H_2_ gas products analysis during OER and HER test. A thermal conductivity detector was used to quantify H_2_ and O_2_ concentration. Chronopotentiometry curves were tested at 10, 20, and 50 mA cm^−2^ for 10, 5, and 2 min, respectively. The gas was collected with syringe with good sealing performance for testing in GC. The calculation of Faraday efficiency is the ratio of actual gas production to theoretical gas production.

## Results and Discussion

### Morphology and Structure Characterization

The cobalt–thiourea coordination complex on NF exhibited the color of purple and became black after power-up (Fig. S1). The evolution curve of the highest temperature on NF during the synthesis processing of NF-C/CoS indicates that the highest temperature of Joule-heating was about 450 °C and the cooling rate was about 100 °C s^−1^ (Fig. S2). The scanning electron microscopy (SEM) and optical micrographs of the cobalt–thiourea coordination complex on NF indicated evenly distributed on NF which was beneficial to the uniform formation of C/CoS (Figs. 1c_1_ and S3).

**Fig. 2:**
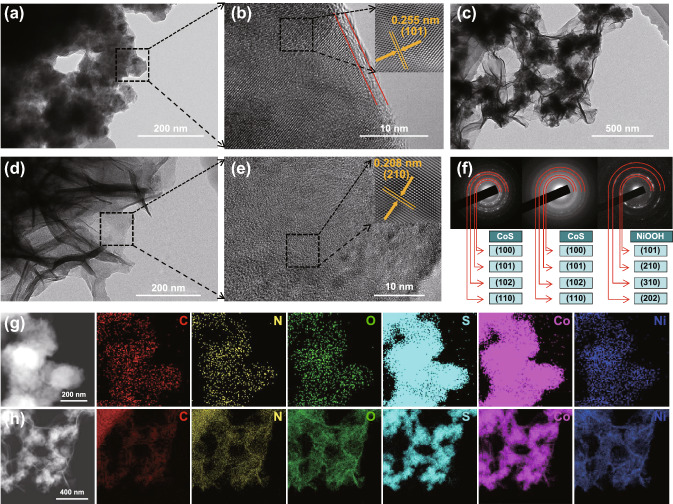
**a** TEM of NF-C/CoS. **b** HRTEM of NF-C/CoS (inset: FFT inverse pattern). **c–d** SEM of NF-C/CoS/NiOOH. **e** HRTEM of NF-C/CoS/NiOOH (inset: FFT inverse pattern). **f** SAED patterns of Fig. 2 **a**, **c,** and **d**, respectively. **g** HADDF image and elemental mapping of NF-C/CoS. **h** HADDF image and elemental mapping of NF-C/CoS/NiOOH

The SEM images of NF-C/CoS showed that carbon-coated CoS nanoparticles grew on the surface of NF (Figs. 1c_2_ and S4a, b). And then the microstructure of NF-C/CoS/NiOOH transforms to hierarchical nanosheets on the surface of C/CoS nanoparticles after soaking treatment of NF-C/CoS in water (Figs. 1c_3_ and S4c, d). The energy-dispersive X-ray spectroscopy (EDS) demonstrated that both NF-C/CoS and NF-C/CoS/NiOOH had C, N, O, S, Co, Ni, and the ratio of Co and S was almost 1: 1 (Fig. S5a, b). Besides, NF-C/CoS/NiOOH showed a sharp increase in oxygen content compared with NF-C/CoS demonstrating the formation of NiOOH by water soaking treatment. The microstructure of C/CoS was irregular particles proved by transmission electron microscopy (TEM) image in Fig. 2a. The high-resolution transmission electron microscopy (HRTEM) image showed crystalline CoS with about 2 nm carbon layer on the surface of CoS marked by red line and the FFT inverse image showed (101) plane of CoS with lattice distance of 0.255 nm (Fig. 2b). Also, the selected area electron diffraction (SAED) pattern proved that the phase of the irregular particles was crystalline CoS with four obvious diffraction rings corresponding to (100), (101), (102), and (110) of CoS, respectively (Fig. 2f) [18]. The elemental mapping showed that the particles were mainly composed of Co and S as well as a small amount of C, N, O (Fig. 2g). The microstructure of C/CoS/NiOOH was core–shell heterostructure with CoS nanoparticles as the core and C/NiOOH hierarchical nanosheets as the shell (Fig. 2c, d). The SAED patterns indicated that the main crystalline phase of C/CoS/NiOOH was still CoS, and the crystalline phase of nanosheets was NiOOH with low crystallinity corresponding to four diffraction rings with (101), (210), (310), and (202) of NiOOH, respectively (Fig. 2f). The HRTEM image showed that the nanosheets contained the amorphous area and the crystalline area which was NiOOH proved by FFT inverse image (Fig. 2e). The elemental mappings indicated that the irregular particles were still CoS and hierarchical nanosheets coated on the CoS included C, N, O, and Ni in which the signal of Ni and O was strong (Fig. 2h). Moreover, the nanosheets were mainly composed of Ni, O and a small amount of C, N, Co, S demonstrating the formation of NiOOH with doped Co and N-, O-, S-doped carbon (Fig. S6). The samples of NF-C/CoS and NF-C/CoS/NiOOH showed complex element composition and structure so that we take the main structure of each part to name in order to simplify the naming of samples. In general, the structure of NF-C/CoS was thin carbon-coated CoS nanoparticles and the structure of NF-C/CoS/NiOOH was C/NiOOH nanosheets on the surface of CoS nanoparticles.

**Fig. 3:**
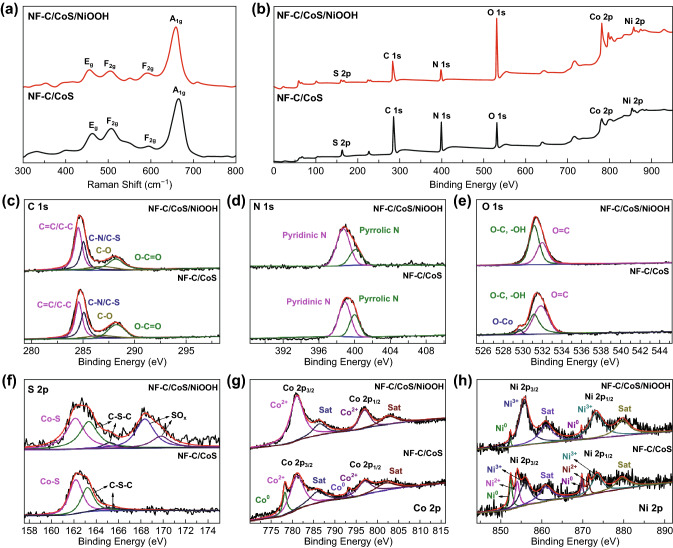
**a** Raman spectrum of NF-C/CoS and NF-C/CoS/NiOOH. **b** XPS survey spectra of NF-C/CoS and NF-C/CoS/NiOOH. **c** C 1 s, **d** N 1 s, **e** O 1 s, **f** S 2p, **g** Co 2p, **h** Ni 2p of NF-C/CoS and NF-C/CoS/NiOOH

The Raman spectra only showed the signal of CoS on NF-C/CoS and NF-C/CoS/NiOOH indicating that the phase of CoS remained stable after soaking treatment in water, and a small amount as well as low crystalline of C and NiOOH (Fig. 3a) [19]. The existence of C, N, O, S, Co, and Ni on NF-C/CoS and NF-C/CoS/NiOOH could also be proved by the X-ray photoelectron spectroscopy (XPS) survey spectra in Fig. 3b. Moreover, the signal strength of O on NF-C/CoS/NiOOH increased compared to NF-C/CoS demonstrating the increase in O after soaking treatment in water. The high-resolution C 1 s spectrum of NF-C/CoS and NF-C/CoS/NiOOH could be deconvoluted into four individual component peaks corresponding to C=C/C–C (284.6 eV), C–N/C–S (285.1 eV), C–O (286.9 eV), and O=C–O (288.3 eV) showing the N-, S-, O-doped carbon (Fig. 3c) [20]. The high-resolution N 1 s spectrum of NF-C/CoS and NF-C/CoS/NiOOH could be proved the formation of pyridinic N (398.9 eV) and pyrrolic N (400.0 eV), as shown in Fig. 3d [21]. The high-resolution O 1 s spectrum could be deconvoluted into three peaks for NF-C/CoS corresponding to C=O (531.8 eV), C–O/–OH (531.2 eV), and Co–O (529.7 eV), but no signal of Co–O could be found in NF-C/CoS/NiOOH (Fig. 3e). The signal strength of C–O/–OH increased showing the formation of NiOOH with –OH [22]. In addition, the signal of Co–O disappeared after soaking treatment in water which was proposed that Co–O came from the bonding of cobalt on the surface of CoS and O on the carbon, and the formation of NiOOH led to the disappearance of Co–O. The high-resolution spectrum of S 2p clearly reflected that both NF-C/CoS and NF-C/CoS/NiOOH had Co–S and C–S–C corresponding to CoS- and S-doped carbon (Fig. 3f) [23]. Importantly, NF-C/CoS/NiOOH showed signal peaks of SO_x_ because of the bonding of S in CoS and O in H_2_O. The high-resolution spectrum of Co 2p clearly reflected that both NF-C/CoS had Co^2+^ as well as Co^0^ and NF-C/CoS/NiOOH only had Co^2+^ that Co^2+^ came from CoS mainly and Co between the surface of CoS and carbon showed Co^0^ due to the reduction of carbon (Fig. 3g) [24]. The high-resolution spectrum of Ni 2p clearly reflected that NF-C/CoS had the signal peaks of Ni^0^, Ni^2+^, and Ni^3+^ that Ni^0^ came from NF and Ni^2+^ as well as Ni^3+^ could come from doped Ni in CoS (Fig. 3h) [25, 26]. And then NF-C/CoS/NiOOH had the signal of Ni^0^ and Ni^3+^ corresponding to NF and NiOOH, respectively.

### Analysis of Formation Mechanism

**Fig. 4:**
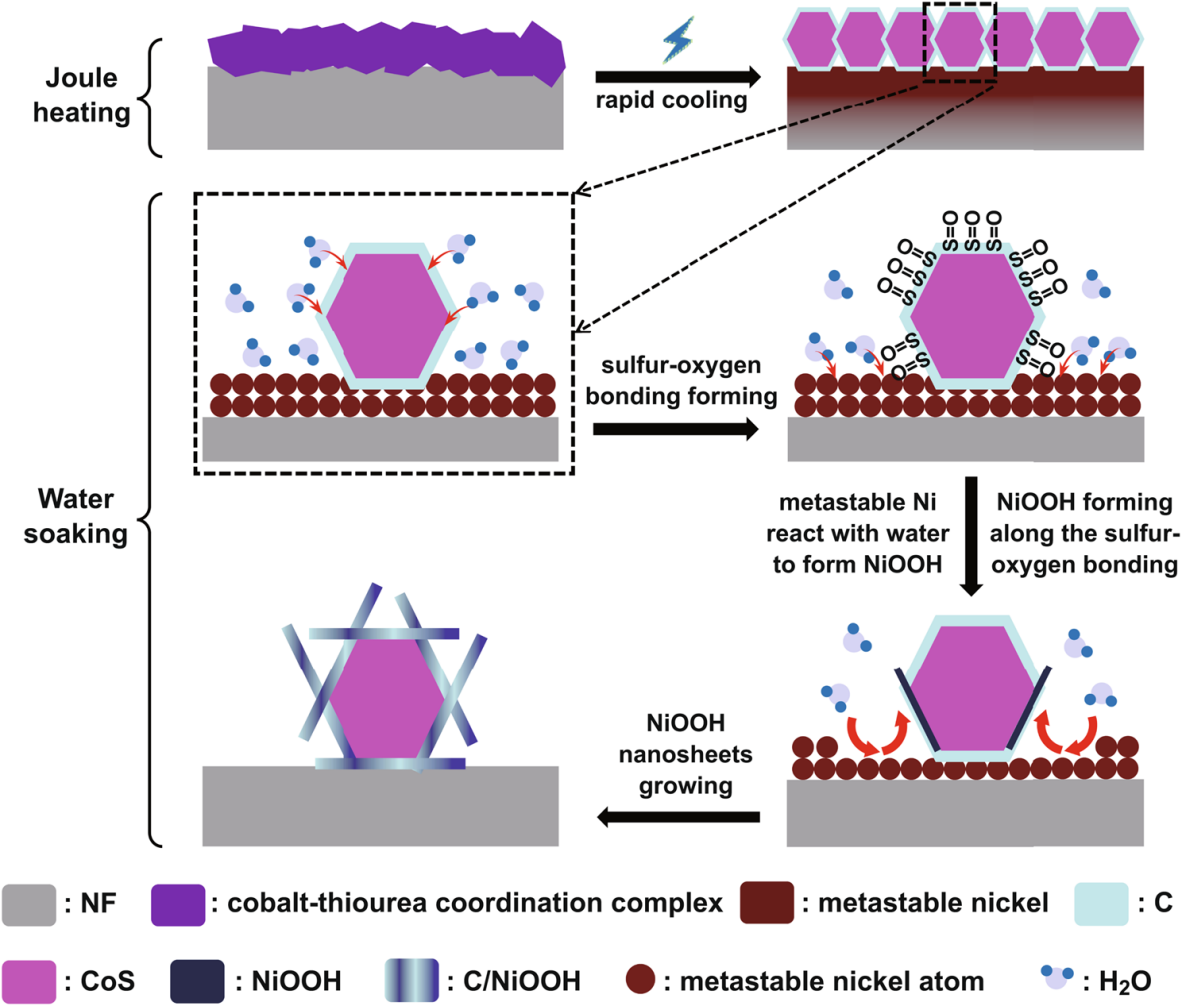
Schematic diagram of the formation mechanism of NF-C/CoS/NiOOH

It was an interesting phenomenon that NiOOH nanosheets grew spontaneously by soaking treatment in water compared with other synthesis methods of nanosheets on NF such as hydrothermal method and electrodeposition method. Here, we made a simple analysis and speculation on the growing mechanism of NF-C/CoS/NiOOH. As shown in Fig. 4, the cobalt–thiourea coordination complex on NF converted to doped carbon-coated CoS by Joule-heating that N-, O-, S-doped carbon came from thiourea and CH_3_COO–. Meanwhile, nickel on the surface of NF was activated to form metastable nickel by cobalt–thiourea coordination complex and Joule-heating. Figure S7 demonstrates that there was no formation of nanosheets on NF-5A indicating that bare NF by only Joule-heating was not activated indicating the necessity of cobalt–thiourea coordination complex for the spontaneous growing of NiOOH nanosheets. When only cobalt acetate solution was added into NF, the as-prepared sample NF-cobalt oxide showed a little cobalt oxide on NF indicating that the activation of nickel was mainly caused by thiourea (Fig. S8). In fact, the S^2−^ of CoS had strong reducibility and instability in water so that S on the surface of CoS could bond with O in water proved in Fig. 3f. In addition, the metastable nickel on the surface of NF reacted with water led to the formation of NiOOH. Moreover, the O which came from water on the surface of CoS became the anchors of NiOOH sustained growing to led to the final formation of C/NiOOH coated on the surface of CoS. Figure S9 shows that the nanosheets of NF-C/CoS/NiOOH-5 min had formed indicating the instantaneity of NiOOH formation. Furthermore, Fig. S10a shows that the crystalline CoS would not form when the current is not high enough (3 A), but it could activate the NF to a certain extent, resulting in a small number of nanosheets. Figure S10b, c demonstrates that NF-C/CoS/NiOOH could be also prepared when the currents were 7 and 10 A higher than 5 A, but the NF would be too brittle to used practically when the current was too high. According to the above data analysis, in general, the driving force of nanosheets structure generation was the activated metastable nickel, metastable nickel reacted with H_2_O to form NiOOH nanosheets, and sulfur–oxygen bonding on the surface of CoS could induce the NiOOH nanosheets growing continually.

### Electrocatalytic Performances

We proved that the activation of nickel was due to the instantaneous thermal effect of Joule-heating and the cobalt–thiourea coordination complex in which thiourea plays an important role. Therefore, we also prepared NF-C/Ni(OH)S with nanosheets structure by the same Joule-heating synthesis and soaking treatment in water for thiourea on NF. There were some similarities in the formation, morphology, and structure for NF-C/CoS/NiOOH and NF-C/Ni(OH)S. It could be proved that the carbon was N-, O-, S-doped carbon and the amorphous Ni(OH)S was and doped by a little Co in NF-C/Ni(OH)S (Figs. S13–S16). However, there were fewer nanosheets structure and no core–shell heterostructure in NF-C/Ni(OH)S compared with NF-C/CoS/NiOOH.

**Fig. 5:**
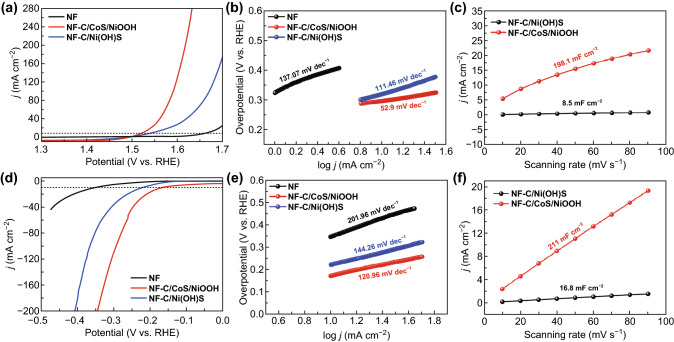
**a** Polarization curves and **b** Tafel slopes of OER on bare NF, NF-C/CoS/NiOOH, and NF-C/Ni(OH)S. **c** *C*_dl_ of OER on NF-C/CoS/NiOOH and NF-C/Ni(OH)S. **d** Polarization curves and **e** Tafel slopes of HER on bare NF, NF-C/CoS/NiOOH, and NF-C/Ni(OH)S. **f** *C*_dl_ of HER on NF-C/CoS/NiOOH and NF-C/Ni(OH)S

The self-supporting electrocatalysts based on NF prepared by the Joule-heating and water soaking treatment could be directly applied to measure the electrochemical performance of OER and HER. The electrochemical performances of OER were evaluated in a three-electrode cell by linear sweep voltammetry (LSV) shown in Fig. 5a. NF-C/CoS/NiOOH exhibited good OER performance with a low overpotential of 296 mV at 10 mA cm^−2^ compared with bare NF (442 mV) and NF-C/Ni(OH)S (319 mV). In addition, NF-C/CoS/NiOOH showed good OER performance with a low overpotential of 361 mV at high current density (100 mA cm^−2^) indicating superior reaction thermodynamics compared with NF-C/Ni(OH)S (449 mV). The electrocatalysts based on NF exhibited a semicircle intersecting with the X-axis of Nyquist plots corresponding to the charge transfer resistance (*R*_ct_) and solution resistance (*R*_s_), respectively. The *R*_s_ related to the impedance of electrolyte depending on Nyquist plots intersection with the X-axis, and the *R*_ct_ related to the current exchange defined by the Butler–Volmer equation depending on the arc diameter of semicircles on Nyquist plots reflecting the intrinsic resistance of electrocatalysts [27]. NF-C/CoS/NiOOH possessed the smallest *R*_ct_ of 3.5 Ω showing a low electronic transport barrier in contrast with bare NF (105.0 Ω) and NF-C/Ni(OH)S (14.0 Ω) (Fig. S17a). Moreover, the smaller Tafel slope of NF-C/CoS/NiOOH (52.9 mV dec^−1^) than bare NF (137.07 dec^−1^) and NF-C/Ni(OH)S (111.46 dec^−1^) indicated its superior OER reaction kinetics and high charge transfer coefficient (Fig. 5b). The ECSA investigated by double-layer capacitance (*C*_dl_) showed that the *C*_dl_ of NF-C/CoS/NiOOH (198.1 mF cm^−2^) was higher than NF-C/Ni(OH)S (8.5 mF cm^−2^) suggesting higher density of catalytical active sites on OER (Figs. 5c and S18a, b). Figure S19a shows the chronopotentiometry curve of NF-C/CoS/NiOOH on OER indicating great stability of OER with an increase of 12 mV after 40,000 s. It was the high activity of NiOOH nanosheets and core–shell heterostructure of C/CoS/NiOOH that led to the great OER performance of NF-C/CoS/NiOOH. Meanwhile, N-, O-, S-doped carbon provides catalytic active sites and fast electron transfer route to some extent. The nanosheets of NiOOH induced and braced by CoS nanoparticles on NF-C/CoS/NiOOH could provide more active site for OER compared with NF-C/Ni(OH)S in which the C/Ni(OH)S nanosheets were only on the surface of NF. In addition, the synergy of NiOOH and CoS core–shell heterostructure could improve the OER activity compared with the homogeneous phase of C/Ni(OH)S.

The electrochemical performances of HER were evaluated in a three-electrode cell by LSV shown in Fig. 5d. NF-C/CoS/NiOOH exhibited good HER performance with a low overpotential of 170 mV at 10 mA cm^−2^ compared with bare NF (347 mV) and NF-C/Ni(OH)S (221 mV) which was similar as the measurement of OER. Moreover, NF-C/CoS/NiOOH showed good HER performance with a low overpotential of 294 mV at high current density (100 mA cm^−2^) compared with NF-C/Ni(OH)S (365 mV). NF-C/CoS/NiOOH also showed the smallest *R*_ct_ of 12.3 Ω similar to the EIS result of OER compared with bare NF (105 Ω) and NF-C/Ni(OH)S (14.9 Ω) demonstrating faster electronic transport on HER measurement (Fig. S17b). The smallest Tafel slope of 120.96 mV dec^−1^ was shown on NF-C/CoS/NiOOH indicating its superior HER reaction kinetics and high charge transfer coefficient in contrast with bare NF (201.96 mV dec^−1^) and NF-C/Ni(OH)S (144.26 mV dec^−1^) in Fig. 5e. Higher *C*_dl_ of NF-C/CoS/NiOOH (211.0 mF cm^−2^) than NF-C/Ni(OH)S (16.8 mF cm^−2^) indicating its higher density of catalytical active sites on HER (Figs. 5f and S18c, d). Figure S19b shows the chronopotentiometry curve of NF-C/CoS/NiOOH on HER that the overpotential of HER was 184 mV after 40,000 s. Similar to the test result of OER, NF-C/CoS/NiOOH showed better HER performance than bare NF and NF-C/Ni(OH)S because of the high activity of NiOOH nanosheets and core–shell heterostructure of C/CoS/NiOOH. The nanosheets structure of NF-C/CoS/NiOOH can maintained after OER and HER (Figs. S21–S25). NF-C/CoS/NiOOH exhibited CoOOH nanosheets and NiOOH nanosheets after OER. NF-C/CoS/NiOOH exhibited S-doped Co(OH)_2_ nanosheets and Ni(OH)_2_ nanosheets.

SEM, EDS, element distribution, and XPS were used to characterize NF-C/CoS/NiOOH after OER test and HER test for 10 h (Figs. S20–S24). NF-C/CoS/NiOOH exhibited CoOOH nanosheets and NiOOH nanosheets after OER. NF-C/CoS/NiOOH exhibited sulfur-doped Co(OH)_2_ nanosheets and Ni(OH)_2_ nanosheets. The carbon doped by N, S, O still exists on nanosheets.

In general, there are three reasons why NF-C/CoS/NiOOH showed good electrocatalytic performance. Firstly, there were more NiOOH nanosheets in NF-C/CoS/NiOOH duo to the induction of CoS compared with Ni(OH)S nanosheets in NF-C/Ni(OH)S. More nanosheets structure can provide more electrocatalytic sites, which can be proved in Fig. 5c, f. Secondly, Studies showed that NiOOH and sulfide forming core–shell structure or heterostructure can provide high OER and HER electrocatalytic activity due to the special structure and synergistic effect of NiOOH and sulfide. After continuous OER and HER test, CoS gradually transformed into CoOOH nanosheets and S-doped Co(OH)_2_ nanosheets, respectively, which still can provide high electrocatalysis activity. Finally, there are much carbon doped by N, O, S in NF-C/CoS/NiOOH and NF-C/Ni(OH)S due to the pyrolysis reaction of cobalt–thiourea coordination complex and thiourea. The carbon doped by N, O, S can provide electrocatalysis activity.

As shown in Fig. S25, the Faraday efficiency results of OER on NF-C/CoS/NiOOH were 93.8% (10 mA cm^−2^), 97.5% (20 mA cm^−2^), and 99.1% (50 mA cm^−2^). The Faraday efficiency results of HER on NF-C/CoS/NiOOH were 95.9% (10 mA cm^−2^), 98.9% (20 mA cm^−2^), and 99.2% (50 mA cm^−2^). In general, NF-C/CoS/NiOOH exhibited high Faraday efficiency at each current density. The Faraday efficiency at high current density is slightly higher than that at low current density because a small part of the current contributes to the pseudocapacitance at low current density.

As shown in Fig. S26a, NF-C/CoS/NiOOH exhibited a good overall water splitting performance with a lower cell voltage of 1.71 V than bare NF (2.04 V) and NF-C/Ni(OH)S (1.81 V) at a current density of 10 mA cm^−2^. Even though the NF-Pt/C//NF-IrO_2_ showed more excellent overall water splitting performance (1.59 V) than NF-C/CoS/NiOOH (1.71 V) at 10 mA cm^−2^, NF-C/CoS/NiOOH exhibited low overall water splitting overpotential (1.96 V) than NF-Pt/C//NF-IrO_2_ (1.97 V) at 200 mA cm^−2^ indicating the great overall water splitting performance at high current density. NF-C/CoS/NiOOH showed smaller *R*_ct_ (11.2 Ω) than bare NF (278.1 Ω) and NF-C/Ni(OH)S (32.9 Ω) which was similar to the EIS test results of OER and HER (Fig. S26b). Figure S26c shows the chronopotentiometry curve of NF-C/CoS/NiOOH on overall water splitting test indicating good stability with the increase of 74 mV after 50,000 s. Figure S26d shows the demonstration of overall water splitting on NF-C/CoS/NiOOH with obvious bubbles generation.

## Conclusions

In summary, we reported a Joule-heating method and water soaking treatment to synthesis the nanomaterials based on NF as self-supporting electrocatalysts fast and simply for overall water splitting. The cobalt–thiourea coordination complex on NF converted to carbon-coated CoS on NF by Joule-heating that the carbon was N-, O-, S-doped carbon and a small amount of Ni was doped into CoS. Notably, the nickel on the surface of NF activated by Joule-heating and cobalt–thiourea coordination complex led to the spontaneous growing of NiOOH nanosheets in water induced by CoS so that NF-C/CoS/NiOOH with hierarchical nanosheets structure and core–shell heterostructure was prepared. We speculated that the driving force of nanosheets structure generation was the metastable nickel and CoS could induce the NiOOH nanosheets growing continually. NF-C/CoS/NiOOH as a self-supporting electrocatalyst exhibited good performance of OER, HER, and overall water splitting. Our work provided a new route to synthesize nanomaterials based on NF that offered a new developing direction in the catalysis and energy field compared with traditional synthesis methods.

## Electronic Supplementary Material

Below is the link to the electronic supplementary material.

Supplementary material 1 (PDF 2487 kb)
