# Supplementary material for: Thermal Shock-Activated Spontaneous Growing of Nanosheets for Overall Water Splitting
Source: Nanomicro Lett. 2020 Aug 12;12:162. doi: 10.1007/s40820-020-00505-2 (PMC7770656; doi:10.1007/s40820-020-00505-2)
Supplement: Supplementary file 1 — Supplementary material 1 (PDF 2487 kb) [file 40820_2020_505_MOESM1_ESM.pdf]

Supporting Information for

Thermal Shock Activated Spontaneous Growing of Nanosheets for Overall Water Splitting

Han Wu[1], Qi Lu[1], Jinfeng Zhang[1], Jiajun Wang[1], Xiaopeng Han[1], Naiqin Zhao[1], Wenbin Hu[1, 2], Jiajun Li[1], Yanan Chen[1, *], Yida Deng[1, *]

[1]School of Materials Science and Engineering, Tianjin Key Laboratory of Composite and Functional Materials, Key Laboratory of Advanced Ceramics and Machining Technology, (Ministry of Education), Tianjin University, Tianjin 300350, People's Republic of China[2]Joint School of National University of Singapore and Tianjin University International Campus of Tianjin University, Binhai New City, Fuzhou 350207, People's Republic of China

*Corresponding authors. E-mail: yananchen@tju.edu.cn (Yanan Chen); yida.deng@tju.edu.cn (Yida Deng)

Supplementary Figures

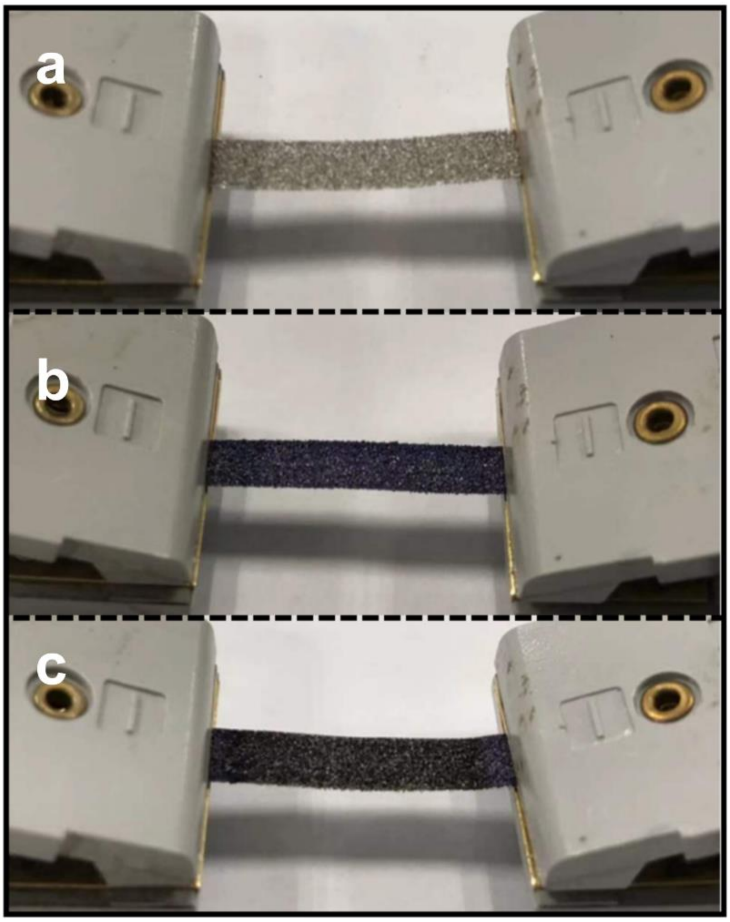

Fig. S1 Practical pictures of **a** bare NF, **b** cobalt-thiourea coordination complex on NF before power up and **c** NF-C/CoS

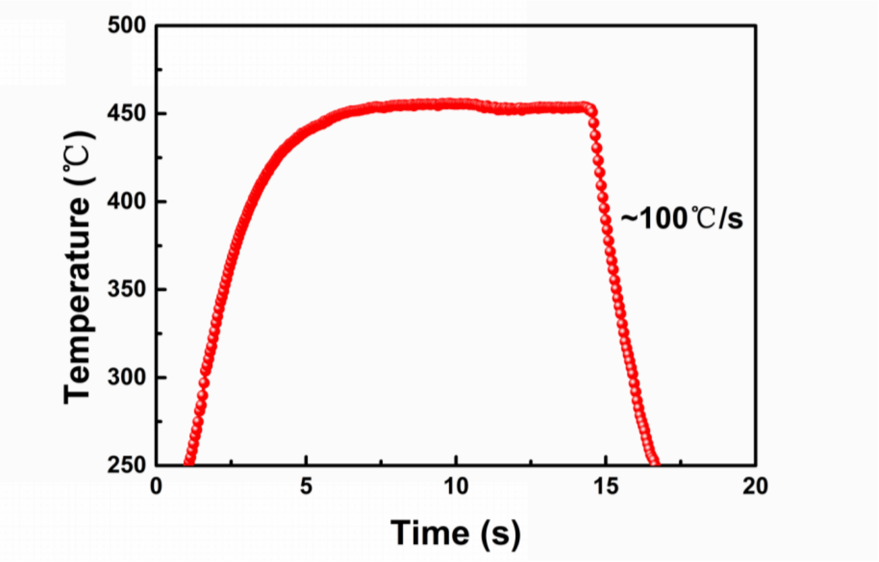

Fig. S2 Highest temperature evolution during synthesis of NF-C/CoS

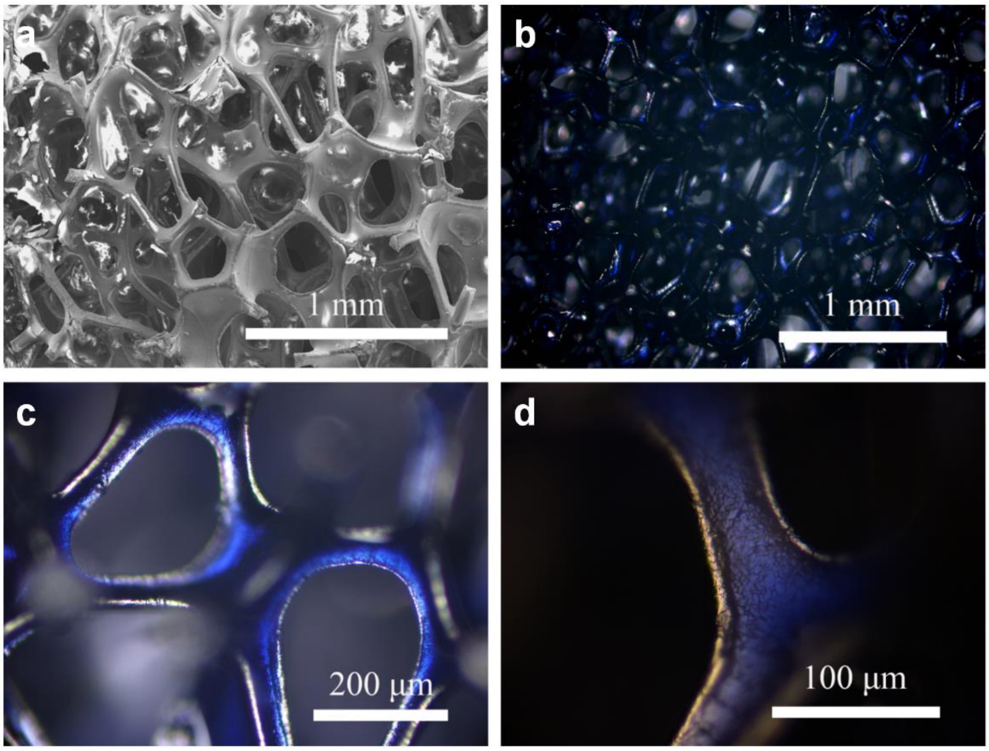

Fig. S3 a SEM images and **c, d** Optical micrographs of cobalt-thiourea coordination complex on NF

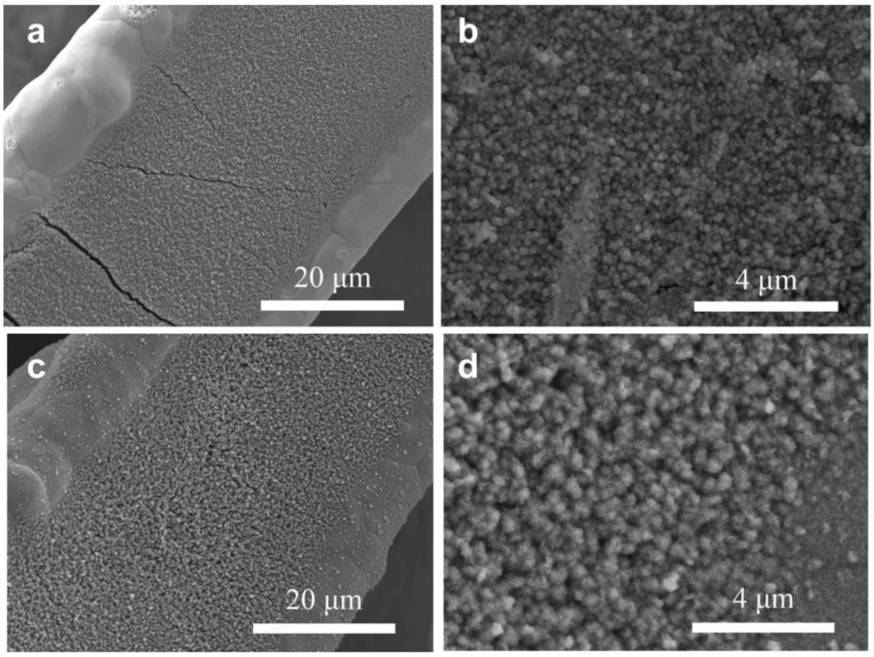

Fig. S4 a, b SEM of NF-C/CoS. **c, d** SEM of NF-C/CoS/NiOOH

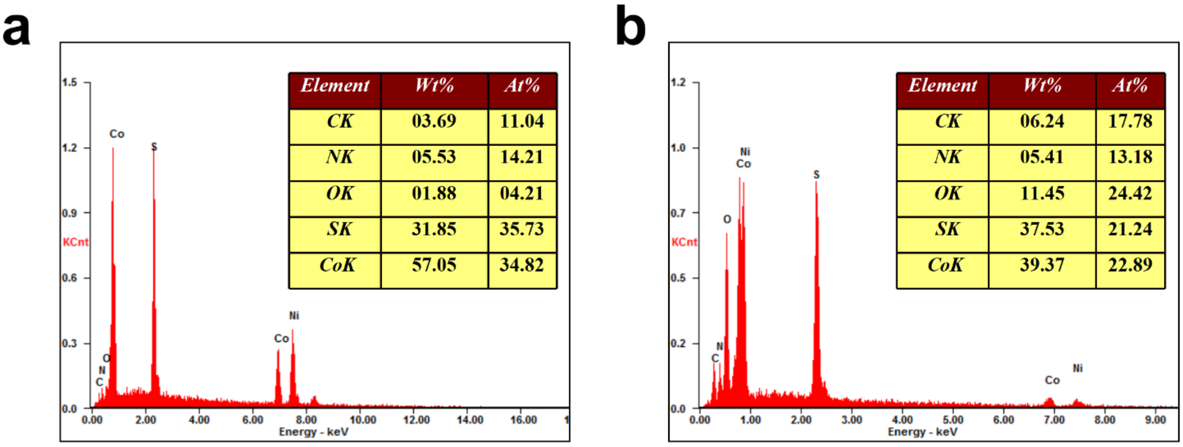

Fig. S5 EDS and element proportion of **a** NF-C/CoS and **b** NF-C/CoS/NiOOH

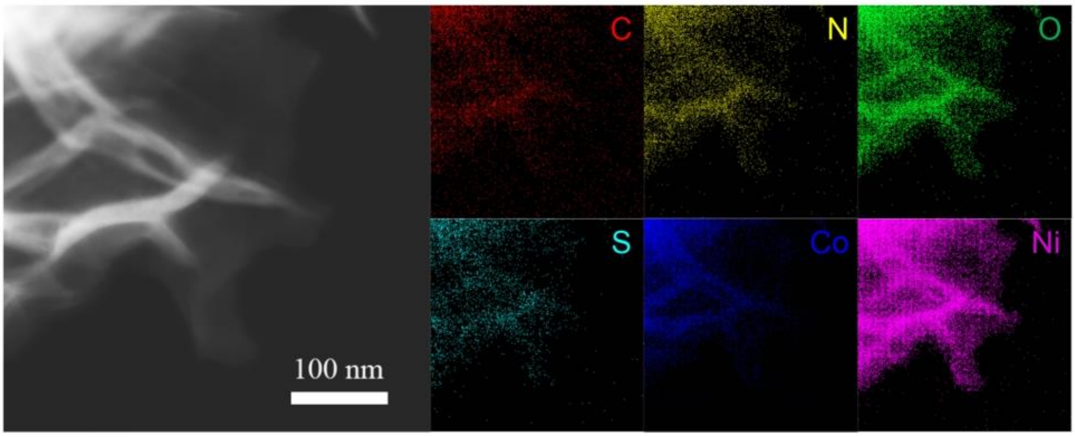

Fig. S6 HADDF image and elemental mapping of partial nanosheets structure on NF-C/CoS/NiOOH

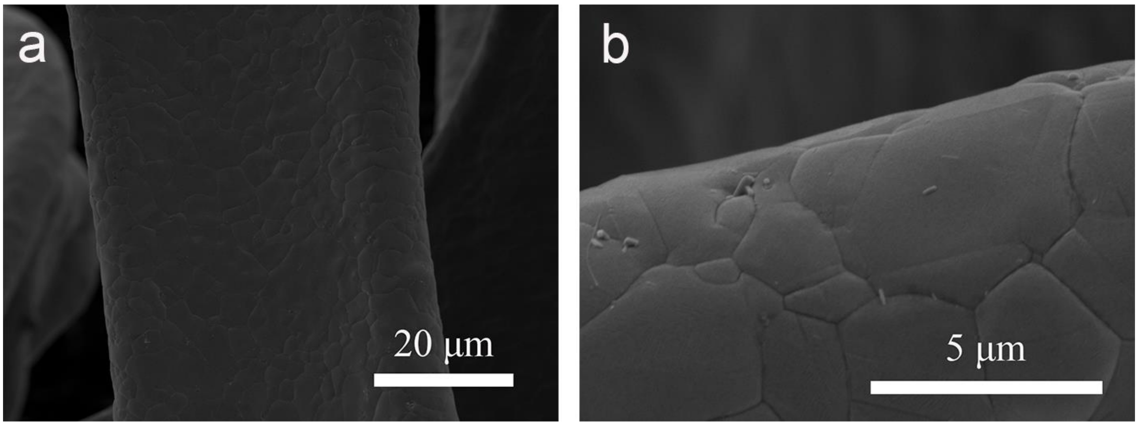

Fig. S7 a, b SEM of NF-5A

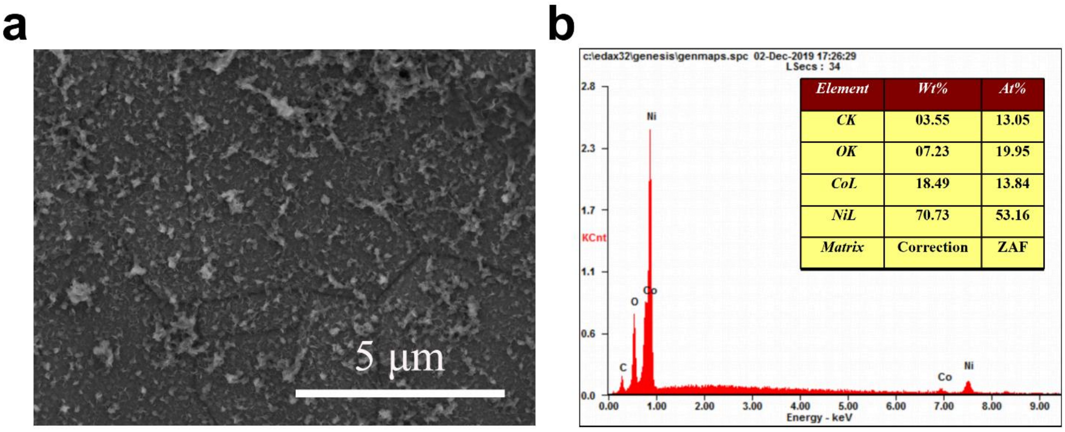

Fig. S8 a SEM of NF-cobalt oxide. **b** EDS and element proportion of NF-cobalt oxide

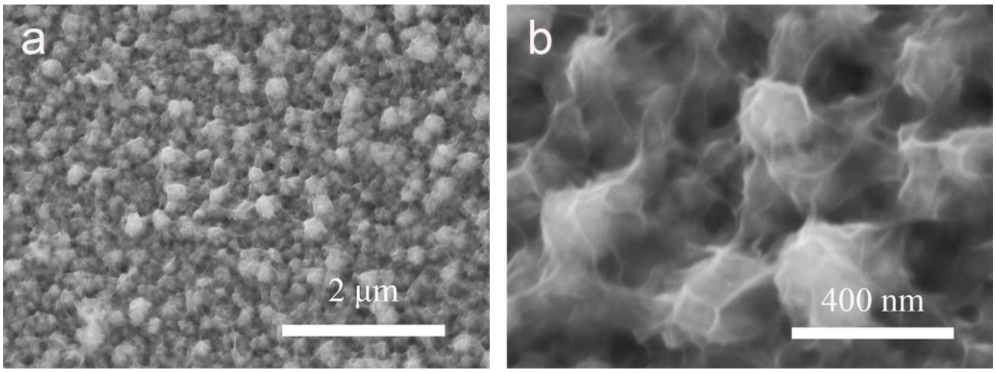

Fig. S9 a, b SEM of NF-C/CoS/NiOOH-5min

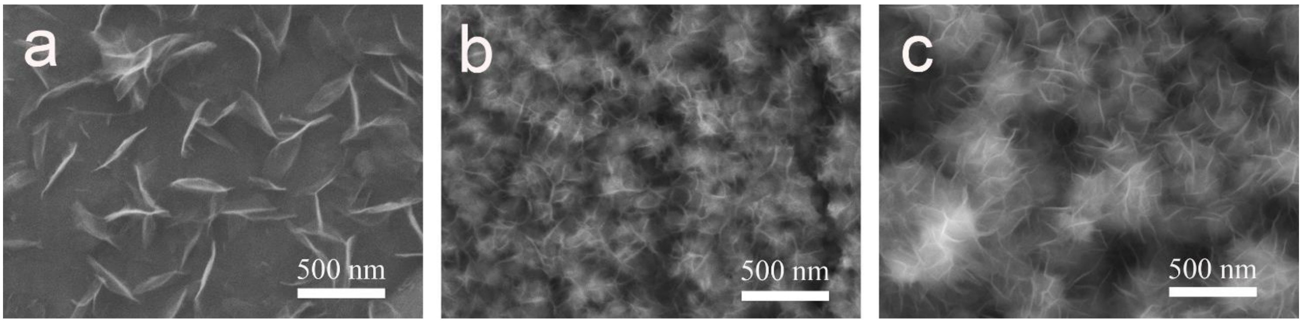

Fig. S10 SEM of **a** NF-C/CoS/NiOOH-3A, **b** NF-C/CoS/NiOOH-7A, and **c** NF-C/CoS/NiOOH-10A

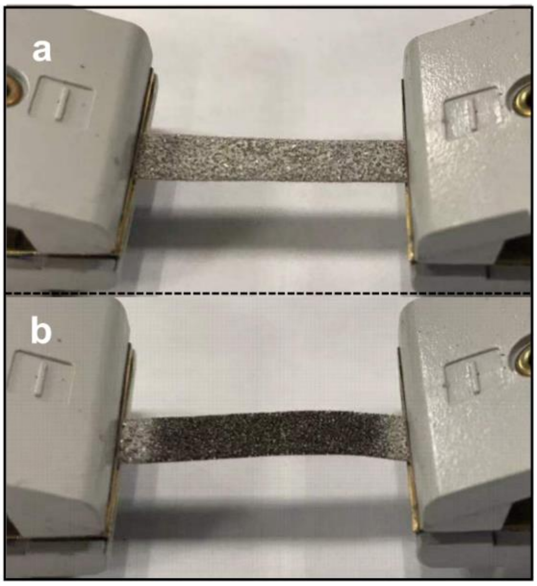

Fig. S11 Practical pictures of **a** thiourea on NF before power up and **b** thiourea on NF after power up at 5A

The practical pictures of thiourea on NF showed transparent thiourea particles on the NF, and then the color became black after power up.

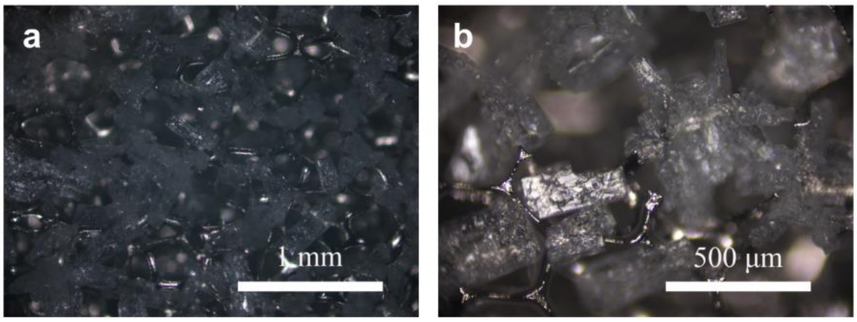

Fig. S12 a, b Optical micrographs of thiourea on NF

The optical micrographs of thiourea on NF showed the hundreds of micrometers thiourea particles

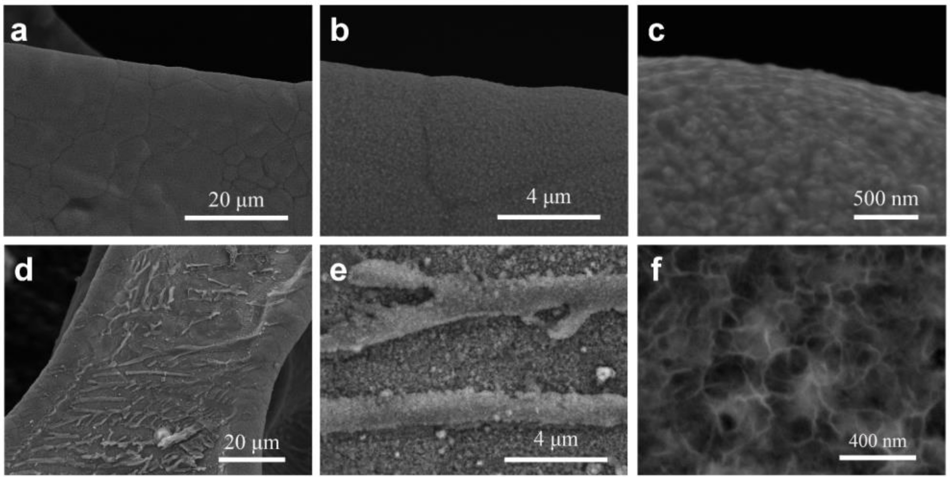

Fig. S13 a-c SEM of thiourea on NF after power up at 5A. **d-f** SEM of NF-C/Ni(OH)S

The SEM images showed that nickel on the surface of NF after power up became rough because the decompostion of thiourea lead to the sulfidation of NF (Fig. S13a-c). After soaking treatment in water, NF-C/Ni(OH)S showed the nanosheets structure similar as NF-C/CoS/NiOOH (Fig. S13d-f). indicating that metastable nickel by activation of thiourea reacted with water leading to the formation of Ni(OH)S.

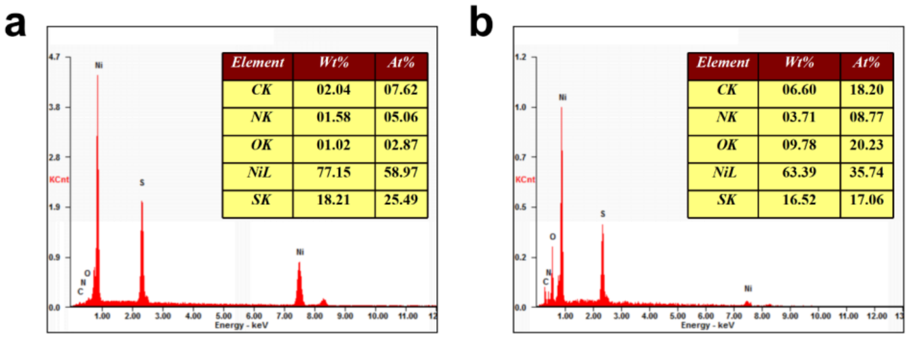

Fig. S14 EDS and element proportion of **a** thiourea on NF after power up at 5A and **b** NF-C/Ni(OH)S

EDS and element proportion demonstrated the existence of C, N, O, S, Ni on NF-C/Ni(OH)S as well as thiourea on NF after power up, and increasing of O after soaking treatment in water indicating the formation of Ni(OH)S.

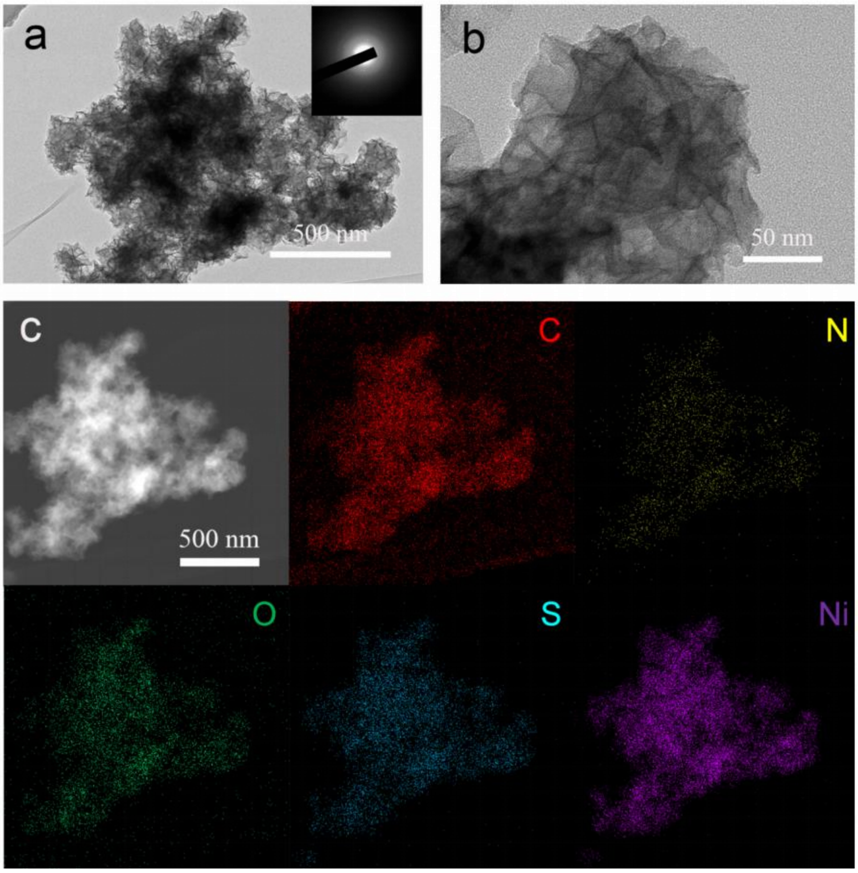

Fig. S15 a TEM of NF-C/Ni(OH)S (insert: SAED pattern). **b** HRTEM of NF-C/Ni(OH)S. **c** HADDF image and elemental mapping of NF-C/Ni(OH)S

The microstructure of NF-C/Ni(OH)S was hierarchical nanosheets (Fig. S15a-**b**. In addition, the

nanosheets of NF-C/Ni(OH)S was amorphous proved by SAED pattern (insert in Fig. S14**a**. Elemental mapping showed the uniform distribution of C, N, O, S, and Ni on NF-C/Ni(OH)S (Fig. S15**c**.

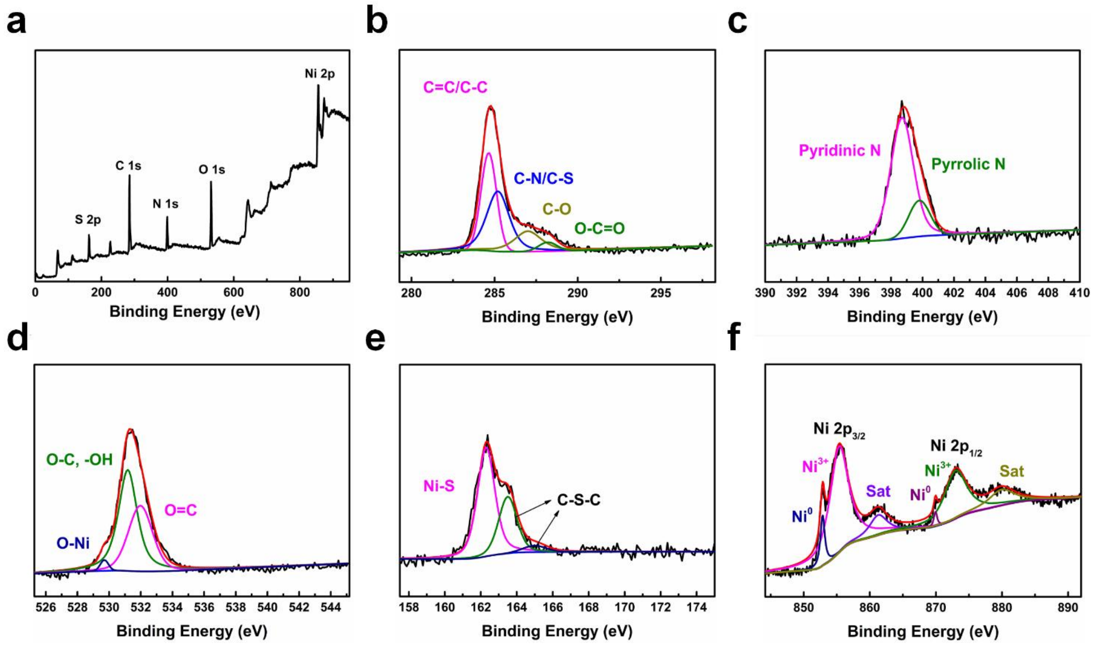

Fig. S16 a XPS survey spectra of NF-C/Ni(OH)S. **b** C 1s, **c** N 1s, **d** O 1s, **e** S 2p, **f** Ni 2p of NF-C/Ni(OH)S

The existence of C, N, O, S and Ni on NF-C/Ni(OH)S can also be proved by the XPS survey spectra similar to NF-C/CoS/NiOOH in Fig. S16a. The high-resolution C 1s spectrum of NF-C/Ni(OH)S showed C=C/C-C (284.6 eV), C-N/C-S (285.1 eV), C-O (286.9 eV), and O=C-O (288.2 eV) indicating O, N, S doped carbon (Fig. S16b). The high-resolution N 1s spectrum of NF-C/Ni(OH)S showed Pyridinic N (398.7 eV) and Pyrrolic N (399.8 eV) in Fig. S16c. The high-resolution O 1s spectrum could be deconvoluted into three component peaks of NF-C/Ni(OH)S corresponding to O=C (531.8 eV), -OH/C-O (531.2 eV) and Ni-O (529.7 eV) indicating the O doped carbon and -OH from Ni(OH)S (Fig. S16d). The high-resolution spectrum of S 2p showed Ni-S from Ni(OH)S and C-S-C from S doped carbon (Fig. S16e). The high-resolution spectrum of Ni 2p clearly reflected that NF-C/Ni(OH)S had the signal peaks of Ni^0 and Ni^{3+} that could come from NF and Ni(OH)S respectively **(**Fig. S16f).

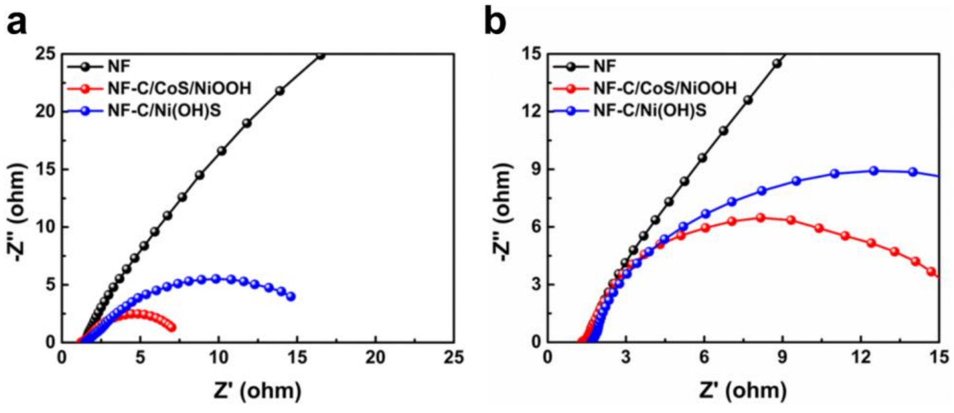

Fig. S17 a EIS of OER on bare NF, NF-C/CoS/NiOOH and NF-C/Ni(OH)S. **b** EIS of HER on bare NF, NF-C/CoS/NiOOH and NF-C/Ni(OH)S

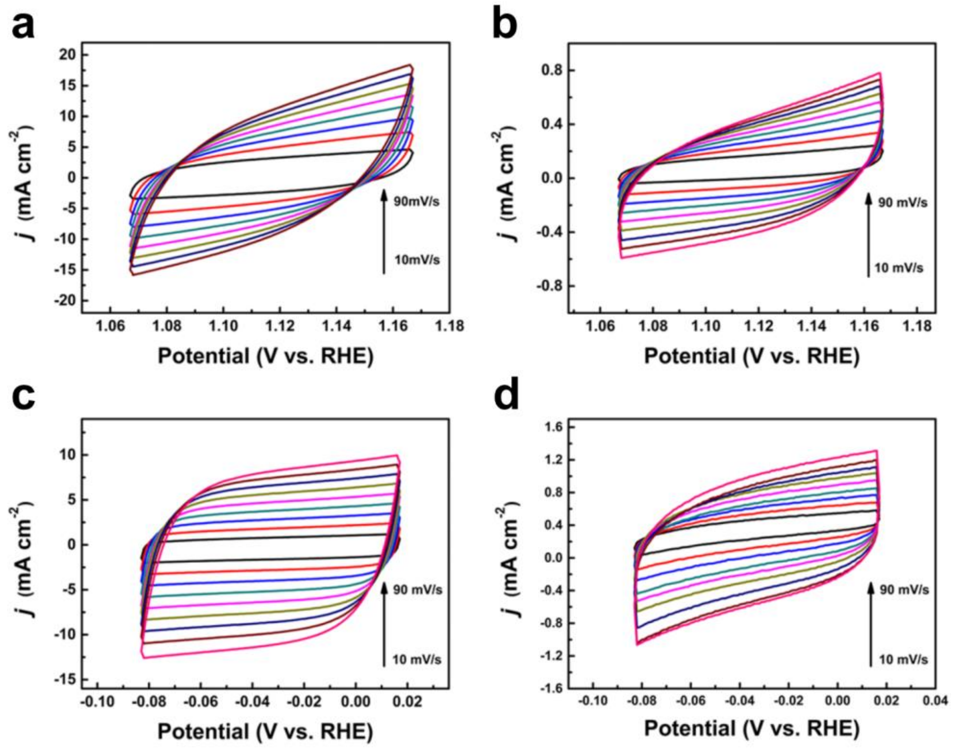

Fig. S18 a, **c** CV curves of NF-C/CoS/NiOOH. **b**, **d** CV curves of NF-C/CoS

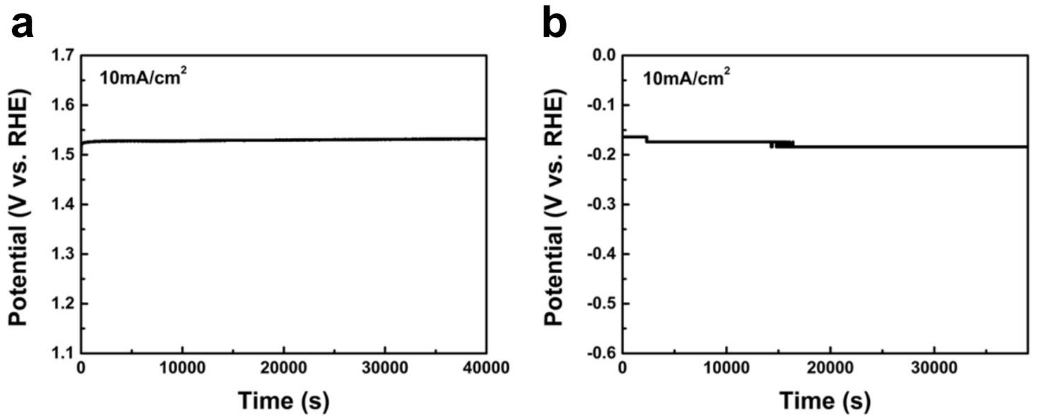

Fig. S19 a Chronopotentiometry curves of OER on NF-C/CoS/NiOOH. **b** Chronopotentiometry curve of HER on NF-C/CoS/NiOOH

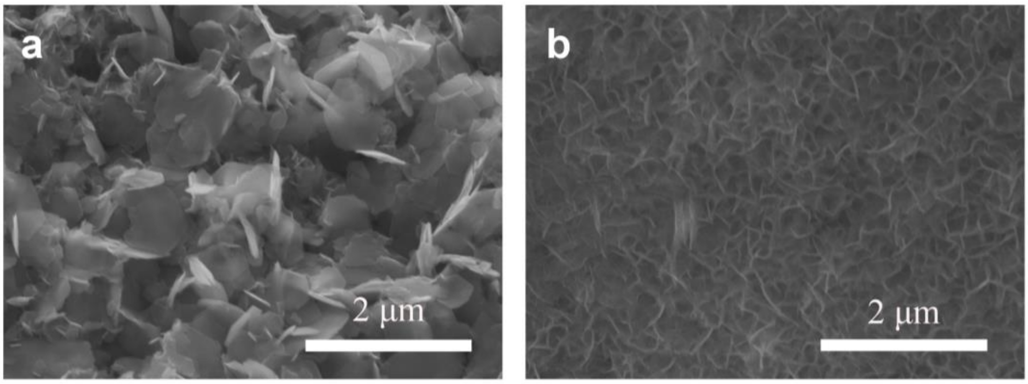

Fig. S20 SEM images of NF-C/CoS/NiOOH after **a** OER and **b** HER

As shown in Fig. S20, NF-C/CoS/NiOOH exhibited nanosheets structure after OER and HER. NF-C/CoS/NiOOH showed smaller interleaved nanosheets and larger hexagonal nanosheets after OER suggesting that the structure of NF-C/CoS/NiOOH maintained nanosheets after OER. NF-C/CoS/NiOOH showed interleaved nanosheets after HER demonstrating nanosheets structure with high active sites still maintained after HER in Fig. S20b.

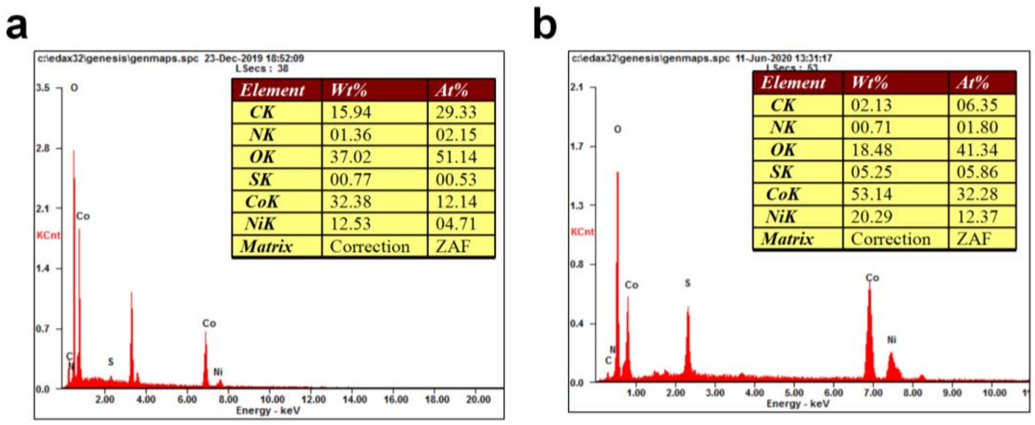

Fig. S21 EDS and element proportion of NF-C/CoS/NiOOH after **a** OER and **b** HER

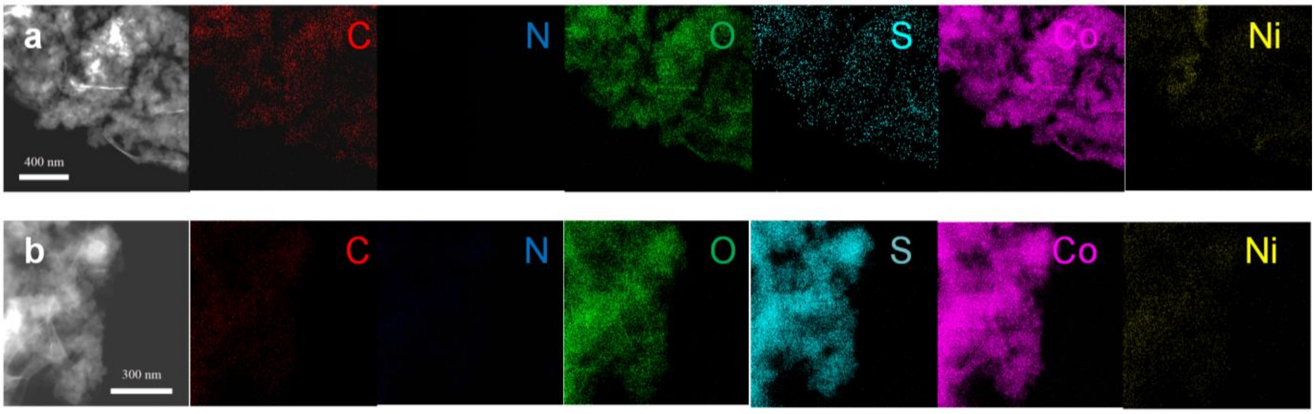

Fig. S22 Element distribution of NF-C/CoS/NiOOH after **a** OER and **b** HER

There were a large loss of sulfur elements on NF-C/CoS/NiOOH after OER because CoS transform to CoOOH under the action of oxidation potential during OER processing in Figs. S21a and S22a. However, CoOOH came from CoS exhibited hexagonal nanosheets which can continue to provide high activity. There was loss of sulfur element on NF-C/CoS/NiOOH which was caused by the intervention of OH- in HER processing in Figs. S21b and S22b. In addition, carbon doped by N, O, S was still evenly distributed with nanosheets structure.

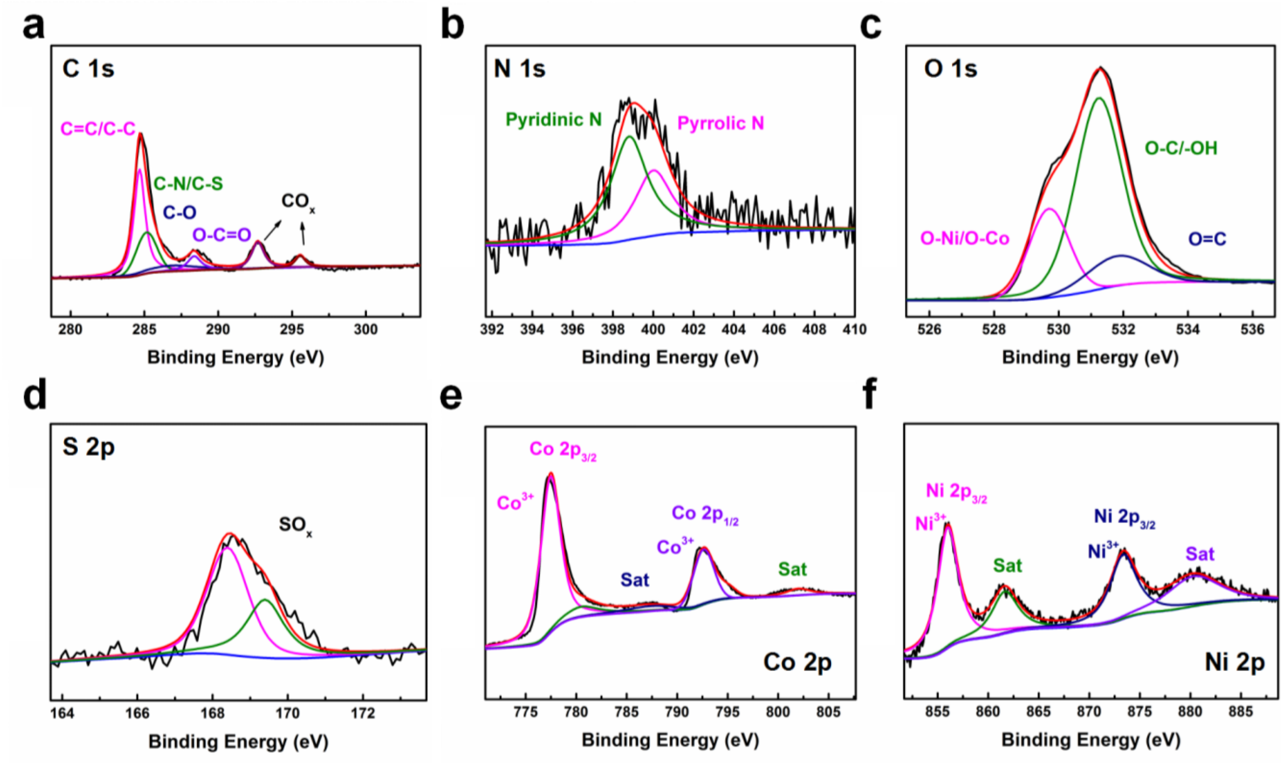

Fig. S23 XPS of NF-C/CoS/NiOOH after OER, **a** C 1s, **b** N 1s, **c** O 1s, **d** S 2p, **e** Co 2p, **f** Ni 2p

There are two new high angle peaks corresponding to CO_x after OER because C was oxidized to form CO_x under the oxidation potential in Fig. S23a. The N was still pyrrole nitrogen and pyridine nitrogen after OER in Fig. S23b. The signal of O element was mainly corresponded to Co-O, Ni-O, -OH after suggesting the forming of CoOOH and NiOOH in Fig. S23c. In addition, Ni and Co were trivalent state corresponding to CoOOH and NiOOH after OER in Figs. S23e, f.

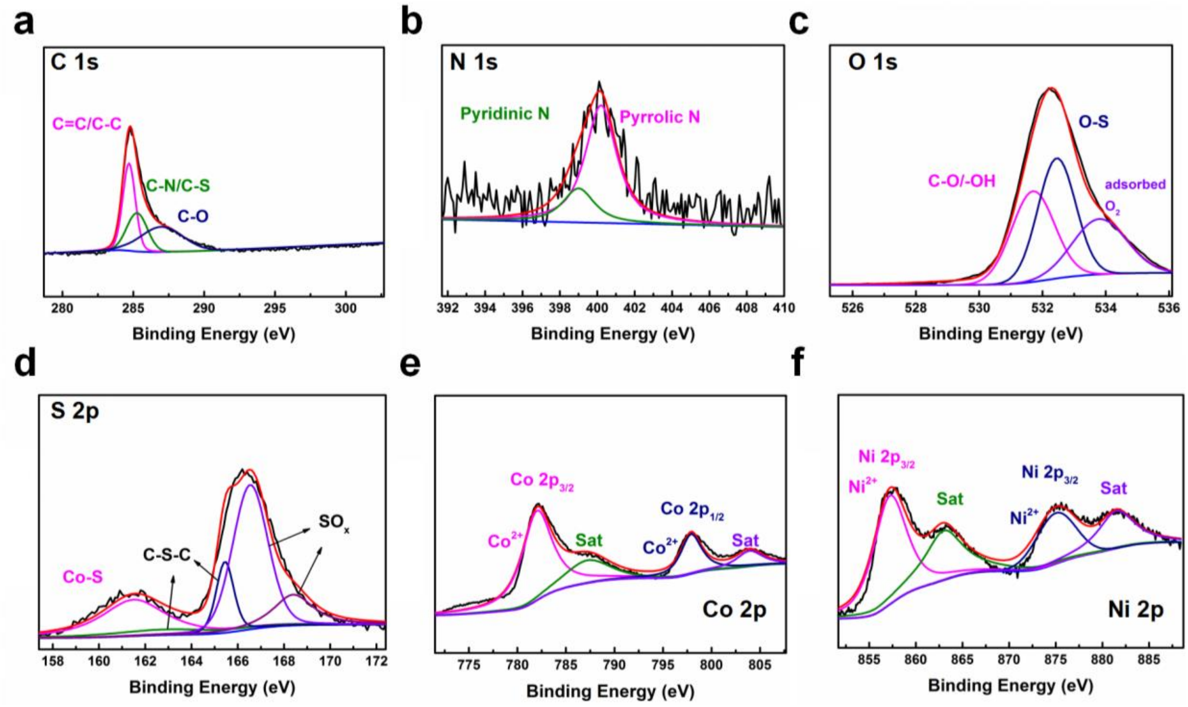

Fig. S24 XPS of NF-C/CoS/NiOOH after HER, **a** C 1s, **b** N 1s, **c** O 1s, **d** S 2p, **e** Co 2p, **f** Ni 2p

The signal of O-C=O disappeared after HER because the carbon with high valence in O-C=O was reduced in Fig. S24a. The N was still pyrrole nitrogen and pyridine nitrogen after HER in Fig. S24b. The presence of f O-S, -OH, Co-S and Co^{2+} proved that CoS transformed into S doped $Co(OH)_2$ during HER (Fig. S24c-e). NiOOH may transformed into S doped $Ni(OH)_2$ in Fig. S24f.

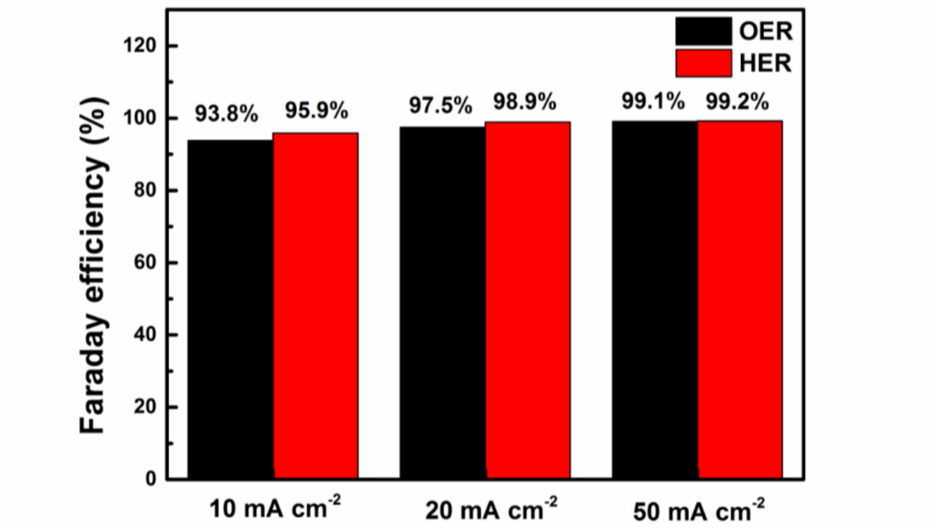

Fig. S25 Faraday efficiency of OER and HER on NF-C/CoS/NiOOH

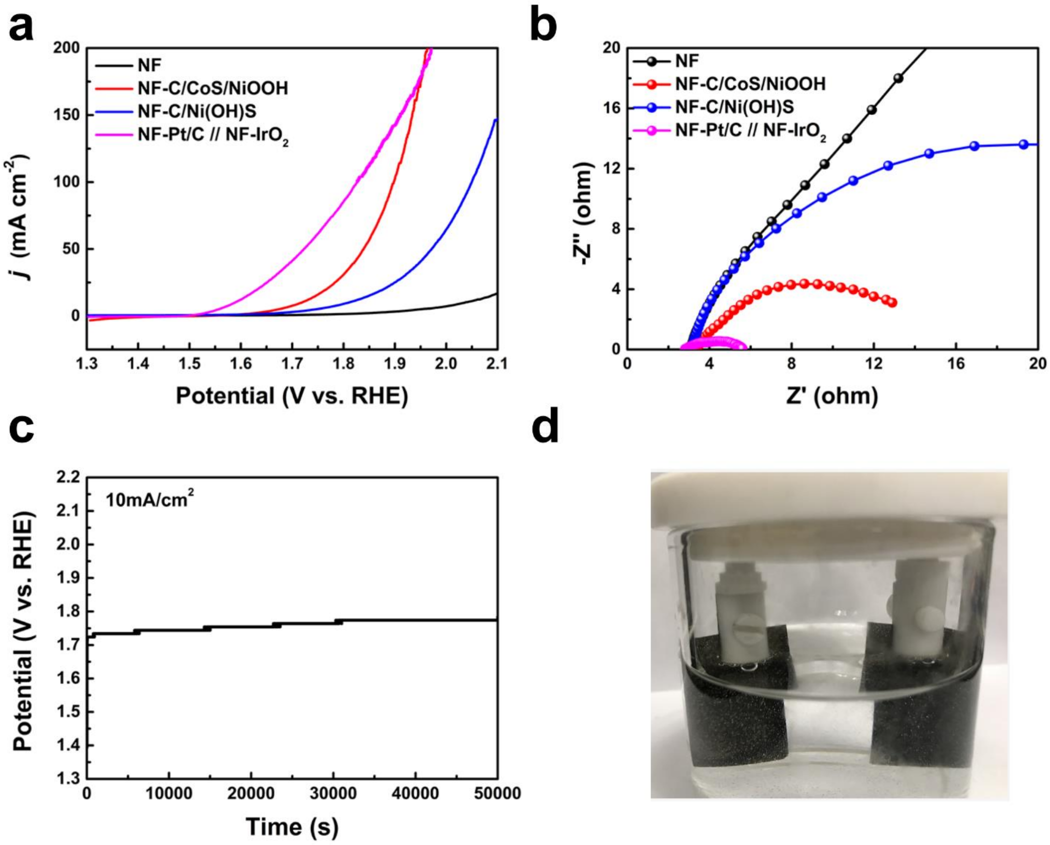

Fig. S26 a Polarization curves of bare NF, NF-C/CoS/NiOOH, NF-C/Ni(OH)S and NF-Pt/C // NF-IrO_2 for overall water splitting. **b** EIS of bare NF, NF-C/CoS/NiOOH, NF-C/Ni(OH)S, and NF-Pt/C // NF-IrO_2 for overall water splitting. **c** Chronopotentiometry curve of NF-C/CoS/NiOOH for overall water splitting. **d** Demonstration of overall water splitting on NF-C/CoS/NiOOH
